# Impacts of environmental and climatic changes on future infectious diseases

**DOI:** 10.1097/JS9.0000000000000160

**Published:** 2023-01-24

**Authors:** Olivier Uwishema, Daniel S. Masunga, Korduni M. Naisikye, Fatemazehra G. Bhanji, Ashley J. Rapheal, Rukia Mbwana, Abubakar Nazir, Jack Wellington

**Affiliations:** a Oli Health Magazine Organization, Research and Education, Kigali, Rwanda; b Clinton Global Initiative University, New York, New York, USA; c Faculty of Medicine, Karadeniz Technical University, Trabzon, Turkey; d Faculty of Medicine, Kilimanjaro Christian Medical University College (KCMUCo), Moshi; e Bugando Medical Centre, Mwanza, Tanzania; f Department of Medicine, King Edward Medical University, Lahore, Pakistan; g Faculty of Medicine, Cardiff University School of Medicine, Cardiff University, Cardiff, UK

Highlights

- Climate changes can influence the pathogens either directly through their effect on their life cycles, breeding, and survival or indirectly by impacting their habitats and environments in which they exist and can bring changes to their ultimate distribution geographically.
- Climate has a great impact on human health ranging from noninfectious to infectious diseases. Climate change influences the transmission of infectious diseases direct and indirect. An indirect effect of climatic change in infectious diseases is through several ways including alteration in biological and ecological processes.
- International organizations in collaboration with other stakeholders in every country should invest more in outbreak responses this will be of vital importance in mitigating the risk of future infectious diseases.

*Dear Editor*,

Environment and climate are generally interdependent that’s the climatic changes haphazardly affect the environment and the environmental changes also affect the climate of a given area. Climate change is a shift in a weather condition with distortion of any of the climate variables which interferes with human health by manipulating survival, reproduction, or distribution of the disease pathogens and hosts^1^. All these changes whether man-made or natural temper human health in a multidimensional context, ranging from changes in simple landscape appearance to changes in human health. Studies have shown diverse effects of both environmental and climatic changes on human health as elucidated below.

Although the impacts of global warming and changes in climate are overlooked, the changes in climatic factors globally are linked to 23% of all death around the globe In 2012 which amounts to 12.6 million death and arises mainly due to contaminated food and water^2^, 34% of all childhood illnesses and 36% of young children’s deaths which are strongly related to outbreaks of cholera, fatal malaria, diarrhea, and dengue fever^3^.

Although climate change is the most influential factor, the level of incidence of infectious diseases concerning climate change has not yet been quantified^4^. Biologically infectious diseases center around the host–pathogen–vector interaction cycle. These environmental and climatic changes tend to temper with either of the components of this cycle leading to an unchecked ecosystem. Studies have shown that when the climate becomes warmer the host–pathogen cycle is disrupted leading to an increased host population, increased pathogen survival, vector range expansion, and hosts may become more susceptible^4^. Other factors such as ozone layer depletion, marine ecosystem disruption, changes in the clean water cycle (due to changes in hydrological systems), and food scarcity may confound to an unstable host–pathogen–vector cycle^5^.

## Environmental and climatic changes in infectious diseases

Environmental factors such as temperature, rainfall, and humidity consequential of climate changes globally pose a significant impact on many infectious pathogens and their patterns^6^. Climate changes can influence the pathogens either directly through their effect on their life cycles, breeding, and survival or indirectly by impacting their habitats and environments in which they exist and can bring changes to their ultimate distribution geographically^1^. There is a considerable prevalence of infectious diseases such as AIDS, neglected tropical diseases, tuberculosis, and malaria and their high mortality and morbidity exist in low and lower-middle-income countries^7^.

Global temperatures are rising at a higher rate than any other time recorded starting from the 1850s and are expected to further increase from 1.8 to 5.8°C at the end of this century. At high temperature, insect vectors are more active for example anopheles’ species of mosquitoes which transmit malaria needs temperature to be greater than 16°C for their life cycles^8^.

Droughts can bring devastating consequences to human health including a rise in infectious diseases, malnutrition, and death which are strongly attributable to unclean drinking water under unhygienic conditions^9^. Droughts and reduced rainfalls cause decreased river flows hence increasing the concentration of water-borne pathogens^1^. While heavy rainfalls and floods following droughts can perpetuate disease transmission to humans when the flood waters are contaminated with human or animal waste causing an increased incidence of fecal–oral transmission of diarrheal and other diseases^10^.

Another significant determinant is absolute humidity, especially in influenza outcomes displaying that decreased humidity caused an increased risk of influenza disease^11^ as well as its influence on other infectious diseases such as dysentery, hand foot and mouth diseases, hepatitis A, hemorrhagic fever, typhoid, malaria, meningitis, and schistosomiasis^12^.

Climate change also brings the possibility of producing asynchrony through disturbance in the availability of hosts required at the time to fulfill the life cycle or via altering the host’s behavior, for example, alteration of fecal excretion change due to abiotic and biotic pressures. This change in synchrony of the circadian rhythm might be able to reduce disease but could also create selection pressure on the parasites to induce changes for a new system^13^. There exists a significant impact of environmental and climatic changes on infectious diseases and it requires global attention.

**Figure:**
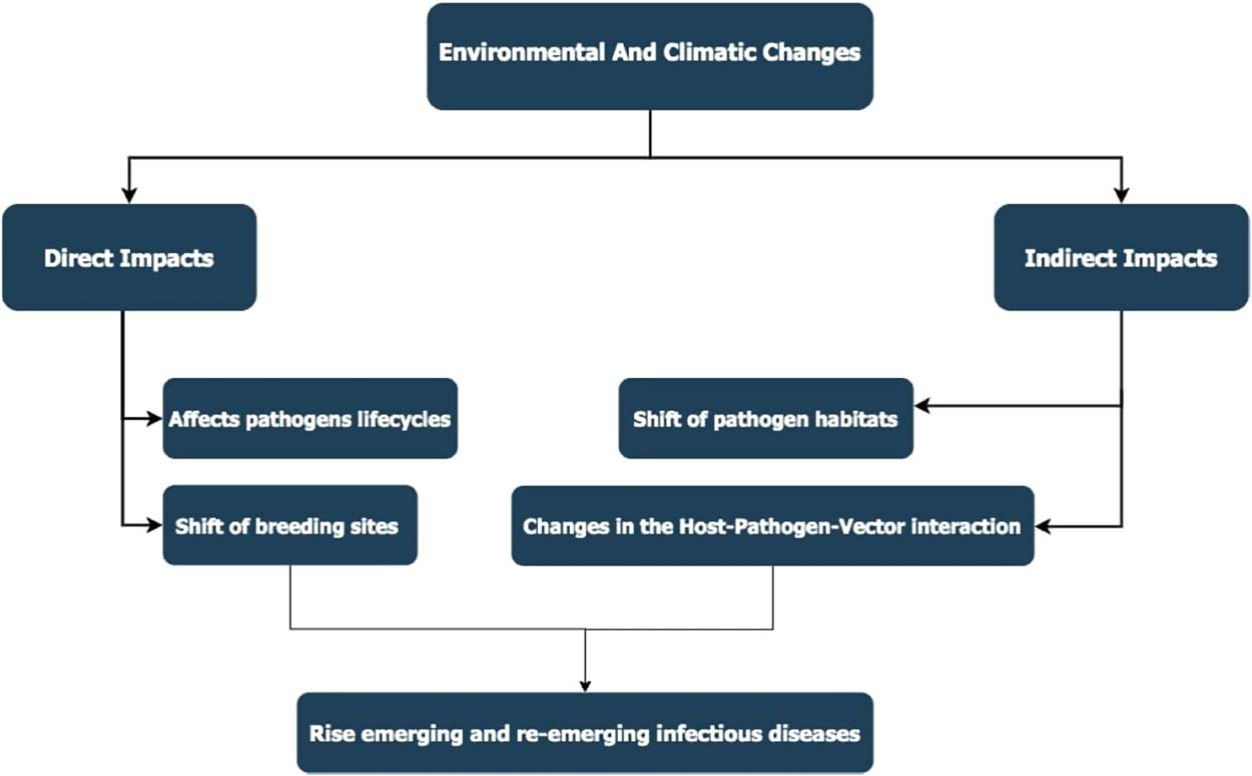


### Factors which trigger climatic changes and its relationship with infectious diseases

Environmental and climatic change is contributed by several factors including human and natural factors^14^. Human activities that can affect climatic changes include those that release harmful gases into the environment. These harmful gases are produced in different ways like deforestation and burning of fossil fuels, especially from industrial activities^15^. Agriculture as part of human activities also has a significant impact on climatic changes as animals release different types of greenhouse gases like methane which is a more powerful greenhouse gas than carbon dioxide^16^.

Climate has a great impact on human health ranging from noninfectious to infectious diseases^17^. Climate change influences the transmission of infectious diseases direct and indirect^18^. An indirect effect of climatic change in infectious diseases is through several ways including alteration in biological and ecological process^17^. Transmission of the infectious disease depends on the pathogen, vector, and host, while all these depend much on environmental and climatic conditions. For instance, most of the vectors that transmit infections depend on climate to regulate their temperature. Hence, any change in the climate that favor the survival of those vectors indirectly influences human health also it can lead to the migration of these reservoir hosts and cause an outbreak of new infectious diseases to areas that favor their survival^19^.

Studies have shown that the incidence of water and vector-borne diseases like malaria and dengue fever are much influenced by environmental and climatic changes^20^. The incidence of water-borne diseases like cholera is said to increase during high and low rainfalls and high temperatures. However, this can also be influenced by other factors like infrastructure, as areas with poor infrastructures are mostly affected by water-borne diseases^21^. But on the other hand, vector-borne diseases like malaria are expected to tip even in wealthier countries due to an increase in the earth’s temperature secondary to global warming^22^. Hence, climatic and environmental changes have a significant impact on infectious diseases and it is something alarming that needs action.

### Infectious diseases in global environmental and climate changes

Infectious diseases may be influenced by several factors as we have seen. However, one factor that has gained widespread global attention is the effect of climate and weather changes on the transmission and prevalence of infectious diseases. For there to be a successful transmission, survival, and reproduction of an organism towards the infection of a human being, three main factors must be considered; the agent (pathogen), the host (vector), and the transmission environment which is where climate change comes into place has divided two ways in which the global climate changes can relate to infectious diseases^1^.

The first one is concerning the host since most animal vectors (hosts) in this regard insects are directly affected by changes in climate. Studies have shown dengue virus, for example, has been significantly affected by temperature changes resulting in increased flying range, increased duration of activity, and shorter extrinsic incubation period^23^.

The second form of development of infectious diseases concerning climate change is a result of indirect transmission of diseases via the responding behavior of humans and vectors to environmental changes^1^. It is the animal’s nature to adapt to environmental changes as we call it the survival of the fittest be it in humans or quite literally animals. These changes in the environment have shown a significant effect on the prevalence of diseases^1^. An increasing prevalence of malaria has been noted concerning temperature changes and rainfall which are all attributed to environmental and climatic changes^24^, while on the contrary infectious diseases like those caused by the Human Influenza virus are most prevalent in conditions associated with wind and dust storms^1^.

Overall, climate changes have been proven to be associated with increasing infectious diseases^23^, and very unfortunately as we are at the peak of global warming, we expect these conditions to only get worse^1^. Climate changes may be used as an essential predictor of disease outbreaks and as a result allow resource-limited settings and developing countries to be able to take necessary precautions and preventive methods but also be well-equipped for a potential outbreak^23^.

### Recommendation

In combating the future emergency and spread of infectious disease pathogens, priorities should be set on reducing inequality to access healthcare services, especially in countries with low resource settings^7^.

Raising public awareness of emerging and re-emerging infectious diseases as the result of environmental and climate changes for its mandatory for the general public to understand the association between increasing health effects due to environmental and climate changes, a better understanding of such association will enable raising awareness, resilience, adaptation, and the improvement of strategies that will safeguard and encourage human health^25^. This can be done by including climate and health content in the school curriculum and continuing education at all levels of education because for a healthy response to environmental and climate change its healthier to have a good understanding of the risks and be part of the solution.

Efforts to emphasize on developing effective and advanced infectious disease surveillance and monitoring systems for emerging and re-emerging infectious diseases in underserved communities of low- and middle-income counties to access epidemic waves and be able to come up with strong international and local policies to tackle the future threats of climatic changes on the rise of infectious diseases^26^.

International organizations in collaboration with other stakeholders in every country should invest more in outbreak responses this will be of vital importance in mitigating the risk of future infectious diseases this can involve efforts to develop a universal vaccine against strains of a zoonotic pathogen (viruses) which have been seen in many of infectious present diseases (like influenza, ebola, coronavirus, and monkeypox diseases)^7^.

Measures to compact environmental pollution by reducing industrial waste production, increasing the use of renewable sources of energy but also enacting strong laws against deforestation will have significant changes to prevent environmental pollution and climatic changes. Improving sanitation conditions by ensuring proper water purification and supply system will be of great deal in preventing the transmission of infectious pathogens to human beings.

**Figure:**
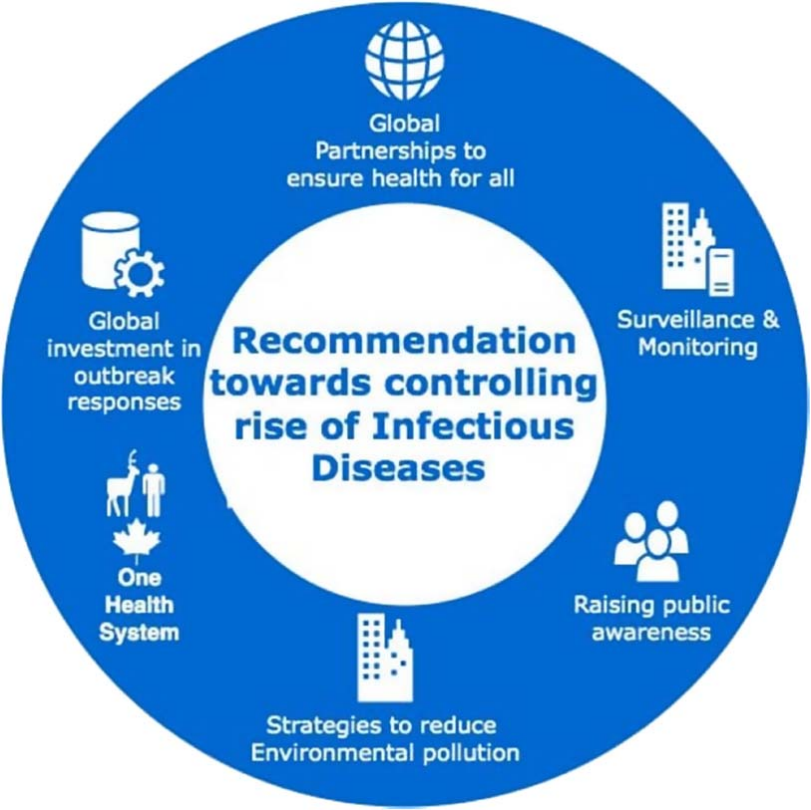


## Conclusion

The recent environmental and climatic changes, particularly the increase in ambient temperature and variation in rainfall amounts, have contributed to the significant changes in the pattern of infectious disease occurrence in various localities around the globe, awareness of the health effects of climate change is not yet widespread therefore it’s important to know that climate change is increasingly affecting health in several ways. Health and public health providers have an important role to play in increasing public awareness of these climate change-associated health risks to human beings. As predictions show that the current trends are expected to continue even more, for better preparedness, any assessment of future transmission of infectious diseases should take into consideration the impacts of climate changes. But also disease and syndromic surveillance implemented at the community level should be part of climate change adaptation measures so that early and quick intervention soon after being identified. Future research should focus to investigate the adverse health impacts of weather and climatic factors in different settings, especially in low- and middle-income countries.

## Ethical approval

Not applicable.

## Sources of funding

None.

## Authors’ contribution

O.U. contributed to the conceptualization, project administration, writing – review and designing. All authors were involved in the manuscript writing, data collection and assembly, final approval of manuscript.

## Conflicts of interest disclosure

The authors declare that they have no financial conflict of interest with regard to the content of this report.

## Research registration unique identifying number (UIN)

Not applicable.

## Guarantor

Abubakar Nazir.

## References

[1] Wu X Lu Y Zhou S . Impact of climate change on human infectious diseases: empirical evidence and human adaptation. Environ Int 2016;86:14–23. 26479830 10.1016/j.envint.2015.09.007

[2] Cissé G . Food-borne and water-borne diseases under climate change in low- and middle-income countries: further efforts needed for reducing environmental health exposure risks. Acta Trop 2019;194:181–188. 30946811 10.1016/j.actatropica.2019.03.012 PMC7172250

[3] El-Sayed A Kamel M . Climatic changes and their role in emergence and re-emergence of diseases. Environ Sci Pollut Res Int 2020;27:22336–22352. 32347486 10.1007/s11356-020-08896-w PMC7187803

[4] Waits A Emelyanova A Oksanen A . Human infectious diseases and the changing climate in the Arctic. Environ Int 2018;121(Pt 1):703–713. 30317100 10.1016/j.envint.2018.09.042

[5] Hall NL Barnes S Canuto C . Climate change and infectious diseases in Australia’s Torres Strait Islands. Aust N Z J Public Health 2021;45:122–128. 33522674 10.1111/1753-6405.13073

[6] Thomas MB . Epidemics on the move: climate change and infectious disease. PLoS Biol 2020;18:e3001013. 33232329 10.1371/journal.pbio.3001013 PMC7685491

[7] Baker RE Mahmud AS Miller IF . Infectious disease in an era of global change. Nat Rev Microbiol 2022;20:193–205. 34646006 10.1038/s41579-021-00639-z PMC8513385

[8] Shuman EK . Global climate change and infectious diseases. N Engl J Med 2010;362:1061–1063. 20335580 10.1056/NEJMp0912931

[9] Funari E Manganelli M Sinisi L . Impact of climate change on waterborne diseases. Ann Ist Super Sanita 2012;48:473–487. 23247142 10.4415/ANN_12_04_13

[10] Dhara VR Schramm PJ Luber G . Climate change & infectious diseases in India: implications for health care providers. Indian J Med Res 2013;138:847–852. 24521625 PMC3978971

[11] Jaakkola K Saukkoriipi A Jokelainen J . KIAS-Study Group. Decline in temperature and humidity increases the occurrence of influenza in cold climate. Environ Health 2014;13:22. 24678699 10.1186/1476-069X-13-22 PMC3978084

[12] Wang Y Rao Y Wu X . A method for screening climate change-sensitive infectious diseases. Int J Environ Res Public Health 2015;12:767–783. 25594780 10.3390/ijerph120100767 PMC4306891

[13] Booth M . Climate change and the neglected tropical diseases. Adv Parasitol 2018;100:39–126. 29753342 10.1016/bs.apar.2018.02.001 PMC7103135

[14] Nwankwoala HNL . Causes of climate and environmental changes: the need for environmental-friendly education policy in Nigeria. J Educ Pract 2015;6:224–234.

[15] Omer AM . Energy, environment and sustainable development. Renew Sustain Energy Rev 2008;12:2265–2300.

[16] Sejian V . Global climate change: role of livestock. Asian J Agric Sci 2011;3:19–25.

[17] Patz JA Githeko AK McCarty JP . Climate change and infectious diseases. Climate Change and Human Health: Risks and Responses. Elseiver; 2003:103–137.

[18] Weiss RA McMichael AJ . Social and environmental risk factors in the emergence of infectious diseases. Nat Med 2004;10(suppl):S70–S76. 15577934 10.1038/nm1150 PMC7095886

[19] Hales S De Wet N Maindonald J . Potential effect of population and climate changes on global distribution of dengue fever: an empirical model. Lancet 2002;360:830–834. 12243917 10.1016/S0140-6736(02)09964-6

[20] Kurane I . The effect of global warming on infectious diseases. Osong Public Health Res Perspect 2010;1:4–9. 24159433 10.1016/j.phrp.2010.12.004 PMC3766891

[21] Hashizume M Armstrong B Hajat S . Association between climate variability and hospital visits for non-cholera diarrhoea in Bangladesh: effects and vulnerable groups. Int J Epidemiol 2007;36:1030–1037. 17664224 10.1093/ije/dym148

[22] Jordan R . How does climate change affect disease. University of Stanford. 2019. Accessed September 2022. https://earth.stanford.edu/news/how-does-climate-change-affect-disease

[23] Hii YL Zaki RA Aghamohammadi N . Research on climate and dengue in Malaysia: a systematic review. Curr Environ Health Rep 2016;3:81–90. 26931438 10.1007/s40572-016-0078-z PMC4789198

[24] Moreira RP Costa AC Gomes TF . Climate and climate-sensitive diseases in semi-arid regions: a systematic review. Int J Public Health 2020;65:1749–1761. 32876770 10.1007/s00038-020-01464-6

[25] Howard C Huston P . The health effects of climate change: know the risks and become part of the solutions. Can Commun Dis Rep 2019;45:114–118. 31285701 10.14745/ccdr.v45i05a01 PMC6587682

[26] No authors listed. Developing infectious disease surveillance systems. Nat Commun 2020;11:4962. 32999286 10.1038/s41467-020-18798-7 PMC7527960

